# Human osteoblasts exhibit sexual dimorphism in their response to estrogen on microstructured titanium surfaces

**DOI:** 10.1186/s13293-018-0190-x

**Published:** 2018-07-03

**Authors:** Michael B. Berger, David J. Cohen, Rene Olivares-Navarrete, Joseph K. Williams, David L. Cochran, Barbara D. Boyan, Zvi Schwartz

**Affiliations:** 1 Department of Biomedical Engineering, College of Engineering, Virginia Commonwealth University, 601 West Main Street, Richmond, VA 23284-3068 USA; 2 Children’s Healthcare of Atlanta, Atlanta, GA 30332 USA; 3 Wallace H. Coulter Department of Biomedical Engineering, Georgia Institute of Technology, Atlanta, GA 30332 USA; 4 Department of Periodontics, University of Texas Health Science Center at San Antonio, San Antonio, TX 78229 USA

**Keywords:** Human osteoblasts, Sex-specific responses, Hormones, Estradiol, 1,25-Dihydroxy vitamin D3, Titanium surface roughness

## Abstract

**Background:**

Osseointegration is dependent on the implant surface, surrounding bone quality, and the systemic host environment, which can differ in male and female patients. Titanium (Ti) implants with microstructured surfaces exhibit greater pullout strength when compared to smooth-surfaced implants and exhibit enhanced osteogenic cellular responses in vitro. Previous studies showed that 1α,25-dihydroxyvitamin D3 [1α,25(OH)_2_D_3_] has a greater effect on rat osteoblast differentiation on microstructured Ti compared to smooth Ti surfaces and tissue culture polystyrene (TCPS). The stimulatory effect of 17β-estradiol (E_2_) on differentiation is observed in female osteoblasts on micro-rough Ti, but it is not known if male osteoblasts behave similarly in response to E_2_ and microtopography. This study assessed whether human male and female osteoblasts exhibit sex-specific differences in response to E_2_ and 1α,25(OH)_2_D_3_ when cultured on microstructured Ti surfaces.

**Methods:**

Osteoblasts from three male and three female human donors were cultured on Ti discs with varying surface profiles: a smooth pretreatment (PT), a coarse grit-blasted/acid-etched (SLA), and an SLA surface having undergone modification in a nitrogen environment and stored in saline to maintain hydrophilicity (modSLA). Cells cultured on these surfaces were treated with E_2_ or 1α,25(OH)_2_D_3_.

**Results:**

Male and female human osteoblasts responded similarly to microstructure although there were donor-specific differences; cell number decreased, and osteocalcin (OCN), osteoprotegerin (OPG), and latent and active transforming growth factor 1 increased on SLA and modSLA compared to TCPS. Female osteoblasts had higher alkaline phosphatase activity and OCN production than male counterparts but produced less OPG. Both sexes responded similarly to 1α,25(OH)_2_D_3_. E_2_ treatment reduced cell number and increased osteoblast differentiation and factor production only in female cells.

**Conclusions:**

Male and female human osteoblasts respond similarly to microstructure and 1α,25(OH)_2_D_3_ but exhibit sexual dimorphism in substrate-dependent responses to E_2_. E_2_ affected female osteoblasts, suggesting that signaling is sex-specific and surface-dependent. Donor osteoblasts varied in response, demonstrating the need to test multiple donors when examining human samples. Understanding how male and female cells respond to orthopedic biomaterials will enable greater predictability post-implantation as well as therapies that are more patient-specific.

**Electronic supplementary material:**

The online version of this article (10.1186/s13293-018-0190-x) contains supplementary material, which is available to authorized users.

## Background

Osteoporosis, osteoarthritis, and spinal disorders disproportionally affect females to a greater extent than males [1]. Traditionally, the imbalance in disease rates between females and males has been attributed to altered levels of circulating sex hormones: estrogen and testosterone. However, there is increasing evidence that the genetic differences in male and female cells result in differences in bone properties, including morphology and size, which contribute to sexual dimorphism between males and females [2]. Extracellular matrix proteins called small integrin binding, *N*-linked glycoproteins (SIBLING proteins), are encoded on the X-chromosome. Mutations in these genes are expressed asymmetrically between males and females, since males inherit only one copy of these genes, and consequently conditions resulting from these gene mutations are more common in boys [3]. Studies have shown mesenchymal stems cells (MSCs) exhibit sexual dimorphism in lineage preference during differentiation. MSCs harvested from male skeletal muscle have an increased affinity to differentiate into chondrocytes versus female cells, and exhibit better cartilage regeneration [4]. Adipose stem cells (ASCs) from post-menopausal females exhibit a greater preference for lineage commitment to osteoblasts than aged-matched male ASCs [5].

Sex differences in bone biology can impact the success of orthopedic and dental implant therapies. It is not known what impact sexual dimorphism at the cellular level has on peri-implant bone formation. Successful osseointegration of implants depends partly on the quality of bone surrounding the implant, as well as systemic circulating factors. In postmenopausal women, estrogen deficiency results in reduced osteoblast activity and increased osteoclast activity, potentially affecting osseointegration. In vivo studies show that in estrogen-deficient female animals, osseointegration around implants is impaired, represented by less bone mass, smaller contact area between bone and the implant [6], and decreased pull-out strength [7]. Although estrogen is also necessary for bone strength in males [8], it is unclear if or how estrogen might affect implant success.

Vitamin D status has been extensively examined with respect to bone health [9]. Vitamin D deficiency is evident in individuals that have limited sun exposure [10–12], and studies have shown that overall fracture healing is hindered in women who are vitamin D deficient [13]. The vitamin D metabolite 1α,25-dihydroxyvitamin D_3_ [1α,25(OH)_2_D_3_] is an important regulator of mineral ion homeostasis, in part by its stimulatory effect on osteoblast differentiation. Studies have shown that the response of osteoblasts to 1α,25(OH)_2_D_3_ in vitro is sensitive to the physical and chemical properties of the substrate, being increased on microtextured titanium (Ti) surfaces compared to smooth Ti surfaces and tissue culture polystyrene (TCPS) [14, 15]. Additional studies have demonstrated a sex-dependent effect of 1α,25(OH)_2_D_3_ on rat osteoblasts grown on Ti implants [16], suggesting that the vitamin D metabolite may affect osseointegration of Ti implants in a sex-specific manner.

Successful osseointegration of an implant is dependent on its surface properties, as the surface is the initial contact point for biological fluids and proteins post-implantation. Ti and Ti alloy implants that possess surface modifications to increase average microroughness and energy result in higher bone-to-implant contact and tighter mechanical integration in vivo than implants with smooth surfaces [17–19]. These results are supplemented by in vitro data in which microtextured surfaces, particularly hydrophilic microtextured surfaces, enhance osteoblast differentiation and osteogenic growth factors, leading to increased matrix deposition [20–23] and soluble factors that decrease osteoclast number and activity, resulting in greater net new bone formation [24].

Sand-blasting followed by acid-etching is a widely used method to create a complex microtopography on Ti. This microstructured surface possesses micro/mesoscale peaks, which are dependent upon acid type, temperature, time, and concentration as well as blasting material, grit size, and pressure [25, 26]. Osteoblasts are sensitive to both micrometer and sub-micrometer scale roughness and are able to discriminate between topographic features [27–29]. Grit-blasting Ti surfaces create 30–100-μm diameter irregular cavities overlaid with 1–3-μm diameter pits, mimicking the morphology of osteoclast resorption pits and providing a biologically analogous surface for osteoblast attachment and new bone formation [30]. Acid-etching creates mesoscale structures, which work synergistically with grit-blasting to control cell adhesion and morphology, and enhance osteoblastic differentiation [31].

Osteoblasts cultured on micro/meso-roughened Ti surfaces create an osteogenic micro-environment by producing more cytokines and growth factors that stimulate osteoblast differentiation while inhibiting osteoclast activity compared to smooth surfaces [32]. These factors can then act in an autocrine/paracrine fashion [33–35]. In addition, osteoblast response to systemically circulating hormones is sensitive to microstructure. MG63 osteoblast-like cells and normal human osteoblasts (NHOsts) have both been shown to be more responsive to 1α,25(OH)_2_D_3_ when cultured on micro-rough surfaces compared to smooth surfaces [14, 15, 36, 37], and this response is robust between species with fetal rat calvarial osteoblasts and mouse osteocyte-like cells behaving similarly [38, 39].

Studies examining human chondrocyte differentiation on microstructured Ti surfaces suggested that there were sex-specific differences in how musculoskeletal cells respond to 1α,25(OH)_2_D_3_ and E_2_. Male chondrocytes were more responsive to 1α,25(OH)_2_D_3_ than female chondrocytes and only female cells responded to E_2_ [40]. This differential behavior was also evident when examining osteoblast response to these hormones in cultures grown on Ti substrates. 1α,25(OH)D_3_ had a more robust stimulatory effect on male rat osteoblast differentiation than on female rat cells [41], but it is not known if this sexual dimorphism also exists in human osteoblasts. Female human osteoblasts responded to E_2_ with enhanced differentiation on the microtextured Ti substrates [42]; however, male human osteoblast response to E_2_ under the same conditions has not been examined.

## Methods

### Preparation and characterization of Ti discs

Fifteen millimeters in diameter Ti discs was prepared from 1-mm-thick sheets of grade 2 Ti as described previously [29, 43–45]. Three surface topographies were used, which have been characterized in detail [21, 44, 46]. Pretreatment discs (PT) are comparatively smooth with an average peak to valley roughness (S_a_) of 0.33 μm with contact angle measurement of 125°. The SLA surface is characterized by uniform peaks and valleys distributed across the surfaces, resulting in a S_a_ of 2.5 μm with contact angle measurement of 135°. The modSLA surface is structurally identical to the SLA surface (S_a_ = 2.7 μm) but has a contact angle measurement of approximately 0°, indicating hydrophilicity. All surfaces were sterilized by gamma irradiation prior to culture.

### Osteoblast cultures

The bone specimens were obtained according to approved Institutional Review Board (IRB) protocols at the Georgia Institute of Technology and Children’s Healthcare of Atlanta (IRB# 05-211). Bone chips were obtained from the iliac crest and ribs from 6 different donors (3 females and 3 males) between the ages of 8 and 16 years during surgery for facial reconstruction. These bone fragments were not needed for the reconstruction and would have been discarded. The fragments were provided without any identifiers other than age and sex, so the puberty status of the donors is not known.

Cells were isolated from the bone fragments using the explant isolation technique [47]. Briefly, each bone specimen was cleaned by removing periosteum and other soft tissues and cut into 1–2-mm pieces. The bone chips were washed three times in Hank’s balanced salt solution (HBSS) containing 3% penicillin-streptomycin. The washed bone chips were digested with 0.25% trypsin-EDTA (Invitrogen, CA) for 15 min at 37 °C. The digestion was discarded to avoid fibroblast contamination. The bone chips were plated in a 100 × 20 Petri dish (BD Falcon, NJ) and cultured in DMEM (Cell Growth, VA) supplemented with 1% penicillin-streptomycin (Invitrogen) and 10% fetal bovine serum (Hyclone, UT). At confluence, the cells were sub-passaged and were plated at 20,000 cells/cm^2^. Confluent third passage cultures were used for the experiments described below.

### Cell isolation characterization

Isolated osteoblasts were first characterized by measuring alkaline phosphatase activity and the level of osteocalcin production in confluent cultures treated for 24 h with or without 10^−8^ M 1α,25(OH)_2_D_3_ treatment for each individual donor, as described below_._

### Cell response

Human osteoblasts were plated at 20,000 cells/cm^2^ density on TCPS, PT, SLA, and modSLA substrates. Media were changed 24 h after plating, with subsequent changes every 48 h till confluence on TCPS. At confluence, the media were replaced with treatment media for 24 h containing vehicle or 1α,25(OH)_2_D_3_ 10^−8^ M (Biomol Research Laboratories, Plymouth Meeting, PA) or 10^−9^ M 17β-estradiol (Sigma-Aldrich Co, St Louis, MO) prior to harvest. TCPS was used as an optical control during culture. Scanning electron microscopy (SEM) imaging was used to confirm cells cultured on PT had a similar morphology to cells on TCPS [27].

Cell number was determined at harvest for all cultures. Cells were released from the surfaces by two sequential incubations in 0.25% trypsin for 10 min at 37 °C, in order to assure that any remaining cells were removed from rough Ti surfaces, and counted using an automatic cell counter (Z1 cell and particle counter, Beckman Coulter, Fullerton, CA). Cells were lysed by freeze-thawing in Triton-X 100. Alkaline phosphatase-specific activity in the lysates was assayed by measuring the release of *p*-nitrophenol from *p*-nitrophenyl phosphate at pH 10.2, and results were normalized to protein content of the cell lysates. The levels of osteocalcin (OCN) in the conditioned media were measured using a commercially available radioimmunoassay kit (Human Osteocalcin RIA Kit, Biomedical Technologies, Stoughton, MA) and normalized by cell number. The conditioned media were also assayed for growth factors and cytokines. Active TGF-β1 was measured prior to acidification of the conditioned media, using an enzyme-linked immunoassay (ELISA) kit specific for human TGF-β1 (TGF-β1 DuoSet, R&D System, Minneapolis, MN). Total TGF-β1 was measured after acidifying the media, and latent TGF-β1 was defined as total TGF-β1 minus active TGF-β1. Osteoprotegerin (OPG) was measured using an ELISA kit (Osteoprotegerin DuoSet, R&D Systems, Minneapolis, MN).

### Statistical analysis

Each variable was tested in six independent cultures in each experiment so that we could statistically compare culture conditions in a single experiment. Data were first analyzed by analysis of variance, when statistical differences were detected; Student’s *t* test for multiple comparisons using Bonferroni’s modification was used. *P* values < 0.05 were considered to be significant. All experiments were repeated to ensure validity, with comparable results. Data comparing the results of male to female (3 patients each) are presented as treatment/control ratios. Statistical significance was calculated using paired Wilcoxon *t* test. *P* values < 0.05 were considered to be significant.

## Results

**Fig. 1:**
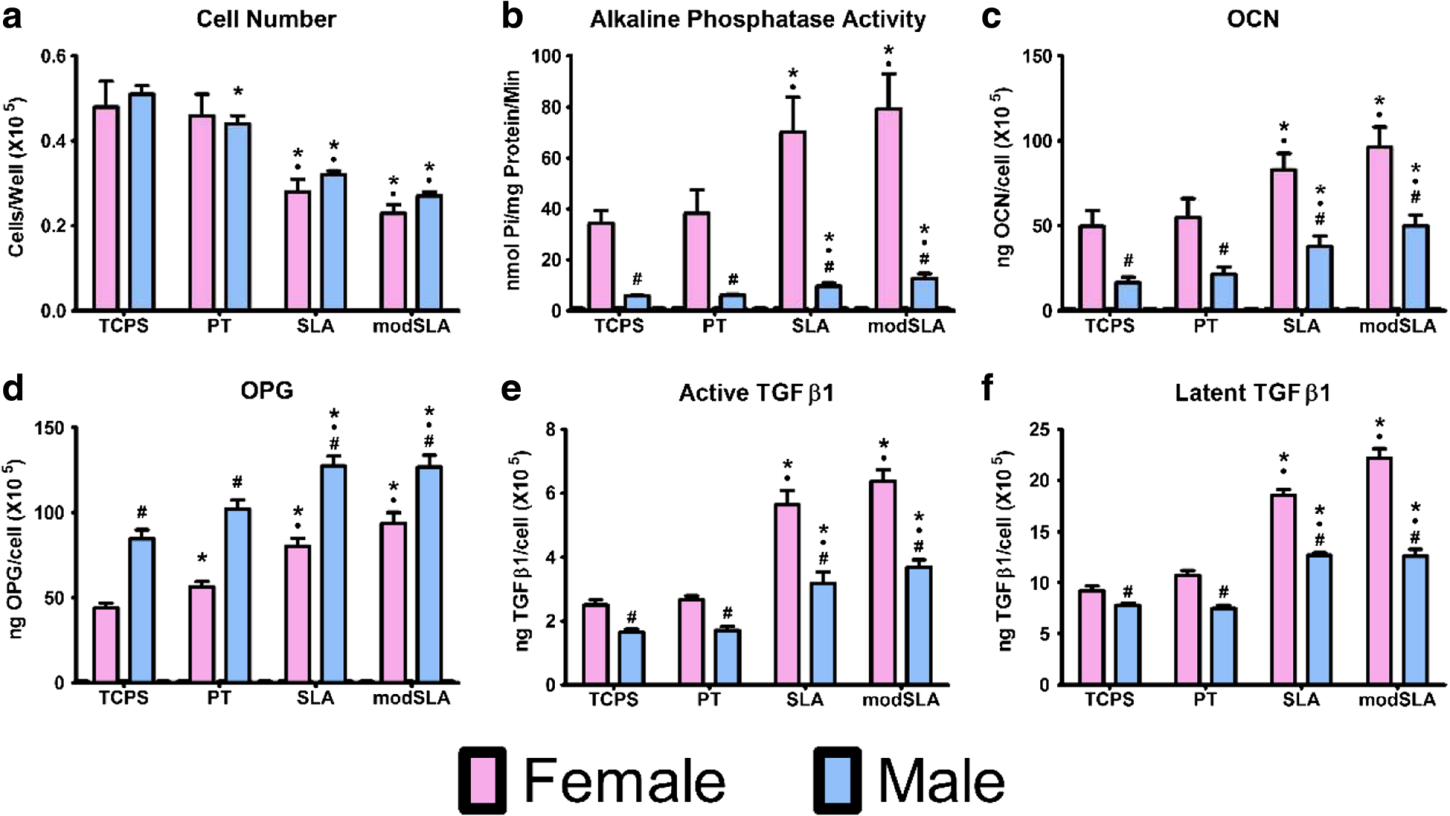
Response of male and female osteoblasts isolated from human donors to microstructured Ti surfaces (PT, SLA, modSLA). Cell number was assessed to determine proliferation of cells, at confluence on TCPS (**a**). Alkaline phosphatase-specific activity was determined in cell lysates (**b**). Production of osteocalcin (**c)**, osteoprotegerin (**d)**, and active (**e**) and latent TGF-β1 (**f**) after 24-h fresh medium incubation. *p < 0.05,vs. TCPS; •*p* < 0.05, vs. PT; ^#^*p* < 0.05, vs. Female cells

Both male and female osteoblasts had surface-dependent reductions in cell number on microtextured Ti (Fig. 1a). Male cells exhibited reduced cell numbers on all Ti substrates compare to TCPS at harvest. The number of cells on SLA and modSLA were comparable and reduced compared to TCPS and PT. Female cells did not exhibit a general Ti-dependent reduction in cell number compared to TCPS, but female cell numbers were reduced on SLA and modSLA, and the effect was comparable to that seen in male cells.

Osteoblast differentiation was sensitive to surface topography. Alkaline phosphatase-specific activity was higher in female cells than in male cells on all substrates (Fig. 1b). Activity was increased by 100% on SLA and modSLA compared to TCPS and PT for both sexes, but the magnitude of the effect was much greater in the female cell cultures. OCN in the conditioned media was affected by the surface in a similar manner (Fig. 1c). Female cells on TCPS and PT produced more than twice as much OCN as male cells. OCN production by male and female cells increased on SLA and modSLA to a similar extent on both surfaces, but again, the magnitude of production was much larger in the female cell cultures.

OPG production was 2 times greater in cultures of male osteoblasts cultured on TCPS than in female cell cultures (Fig. 1d). Male osteoblasts increased production on all Ti surfaces, with the greatest effect on SLA and modSLA, but the increase was never greater than the levels of OPG produced by female cells on PT surfaces. Female osteoblasts produced more OPG on SLA than on TCPS and PT, but the robustness of the increase was not as great as seen in male cells. In contrast to production of OPG, media from male cell cultures had less active and latent TGF-β1 than from female cell cultures on all surfaces (Fig. 1e, f). Levels doubled in male cultures on SLA and modSLA, but there was almost a threefold increase in both active and latent TGF-β1 in media from female cultures on these surfaces compared to TCPS. Active TGF-β1 was sensitive to surface topography in female and male cultures, with greater production on SLA and modSLA than on TCPS and PT (Fig. 1e). Male cells also exhibited greater donor to donor variability in response to the surface topography (Additional file 1: Figure S1 and Additional file 2: Figure S2).

**Fig. 2:**
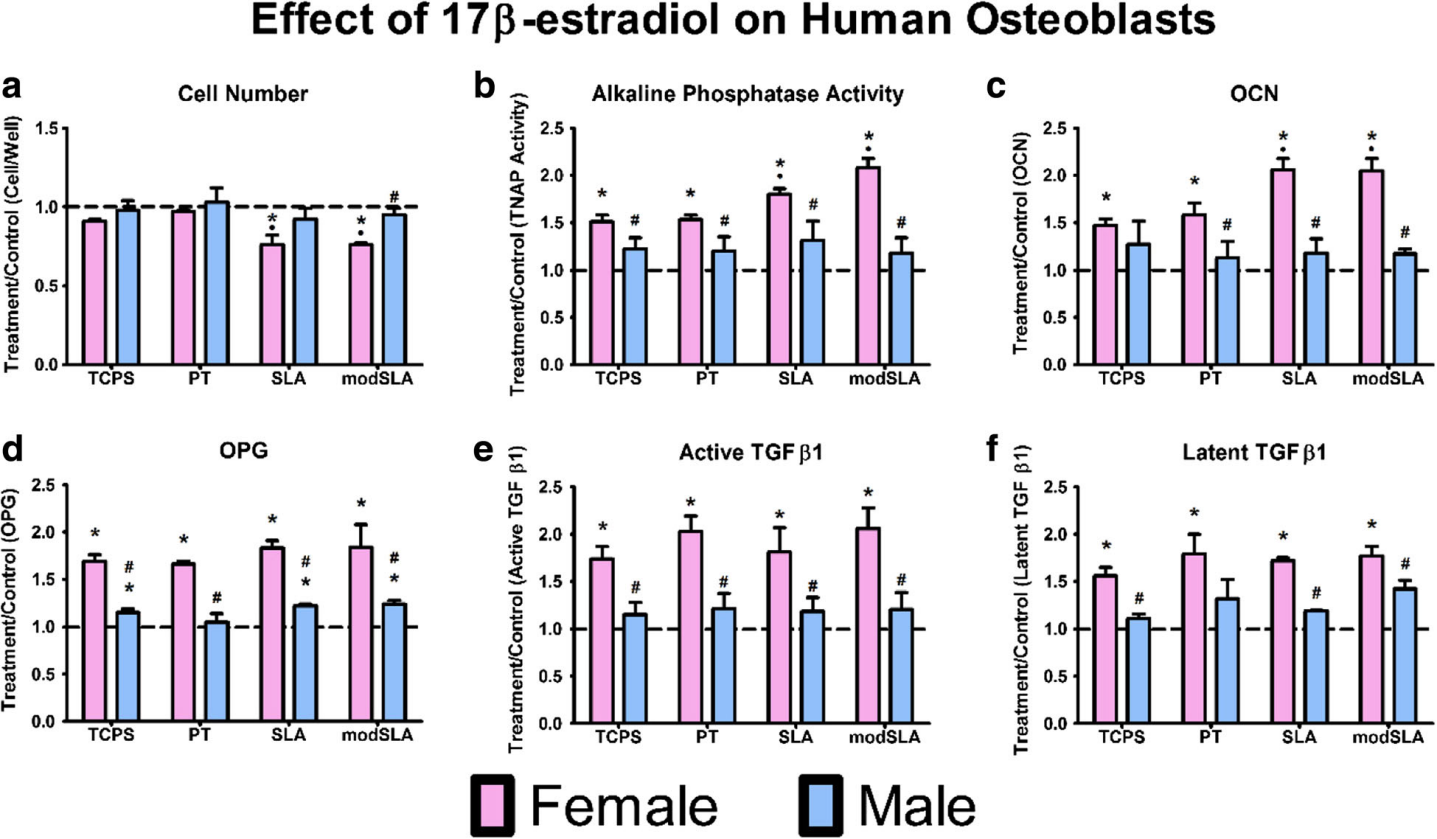
Treatment over control for male and female cells cultured on microstructured Ti surfaces and treated with 17β-estradiol for 24 h at confluence on TCPS. Cell number (**a)** and alkaline phosphatase-specific activity in cell lysates was assessed (**b**). Production of osteocalcin (**c**), osteoprotegerin (**d**), and active (**e**) and latent TGF-β1 (**f**) after 24-h fresh medium incubation. *p < 0.05, vs. untreated control per surfaces; •*p* < 0.05, vs. PT; ^#^*p* < 0.05, vs. female cells

E_2_ had no effect on cell number in either female or male cells cultured on TCPS or PT (Fig. 2a). While E_2_ caused reduced cell number in female cells on SLA and modSLA compared to control cells on these surfaces, it had no effect on male cell cultures. Alkaline phosphatase specific activity was increased in all female cells grown on all surfaces, with the greatest stimulatory effect of the hormone in female cells grown on SLA and modSLA (Fig. 2b). There was no effect of the hormone on male cells on any surface compared to control cultures. Similarly, E_2_ stimulated OCN production in female cells on all surfaces with the greatest effect on SLA and modSLA, whereas E_2_ had no effect on male cells relative to control cultures (Fig. 2c).

E_2_ caused a marked increase in production of OPG on all surfaces compared to control cultures, but there was no surface-dependent effect (Fig. 2d). Male cells exhibited a small but significant increase in production of OPG on TCPS, SLA, and modSLA. This response to the hormone was much less robust than the response observed in female cultures. Similarly, E_2_ increased the levels of latent TGF-β1 in female cell cultures on all surfaces to a comparable extent (Fig. 2f). Levels of this growth factor in the conditioned media of male osteoblast cultures were unaffected by treatment with E_2_.

**Fig. 3:**
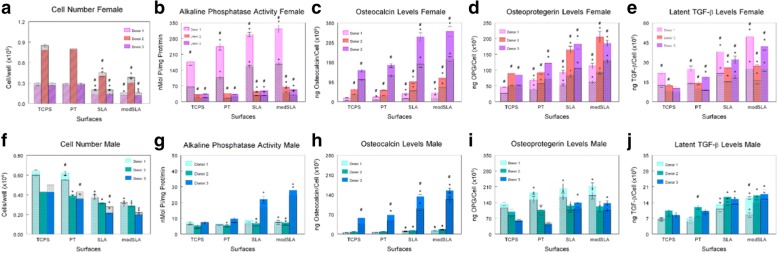
Donor-specific response of male and female cells cultured on microstructured Ti surfaces and treated with 17β-estradiol for 24 h at confluence on TCPS. Cell number was assessed to measure proliferation (**a**, **f**). Alkaline phosphatase-specific activity in cell lysates was assessed (**b**, **g**). Production of osteocalcin (**c**, **h**), osteoprotegerin (**d**, **i**), and latent TGF-β1 (**e**, **j**) after 24-h fresh medium incubation. *p < 0.05, vs. TCPS; ^#^*p* < 0.05, vs. untreated control group per surface

Response to E_2_ varied with the donor for both male and female osteoblasts (Fig. 3). E_2_ had an inhibitory effect on female cell number on all surfaces, but the extent of the reduction varied by surface and by the donor (Fig. 3). E_2_ had a stimulatory effect on alkaline phosphatase activity in all female cell cultures, but the sensitivity to the surface varied among donors (Fig. 3b). OCN and OPG exhibited comparable fold increases in response E_2_ in all female cell cultures, but both proteins exhibited the greatest increases in control on SLA and modSLA resulting in the greatest overall levels in the treated cultures on those surfaces (Fig. 3c, d). Active TGF-β1 was increased by 100% on all surfaces after E_2_ treatment for female donors, while male cells either exhibited no change in response to the hormone or had small changes in production, which were not statistically significant (Additional file 1: Figure S1). Both sexes produced more latent TGF-β1 than active growth factor (Fig. 3e, j; Additional file 1: Figure S1). Latent TGF-β1 was higher in E_2_-treated female cultures on all surfaces (Fig. 3e). Not all donors responded to E_2_ on TCPS, but the stimulatory effect of E_2_ on the Ti substrates was comparable in terms of fold increase, although donor variability in control levels resulted in considerable variation in outcome.

Male cells exhibited donor variability for all parameters (Fig. 3f–j). Some effect of E_2_ was observed, but there was no overall pattern. Donor 1 and donor 3 exhibited E_2_-dependent changes in cell number, but donor 1 had an increase in cell number on PT whereas donor 3 had a decrease on PT. Similarly, donor 1 exhibited a small but significant increase in enzyme activity on SLA whereas donor 3 exhibited a small increase on SLA and modSLA. OCN production was elevated in donor 3 cultures on all surfaces. E_2_ stimulated OPN in donor 1 cultures, and latent TGF-β1 was increased in donor 1 cultures grown on Ti substrates with the greatest effect on modSLA. Donor 1 exhibited no E_2_-dependent increase in active TGF-β1, whereas E_2_ caused a small increase in active growth factor by cells from donors 2 and 3.

**Fig. 4:**
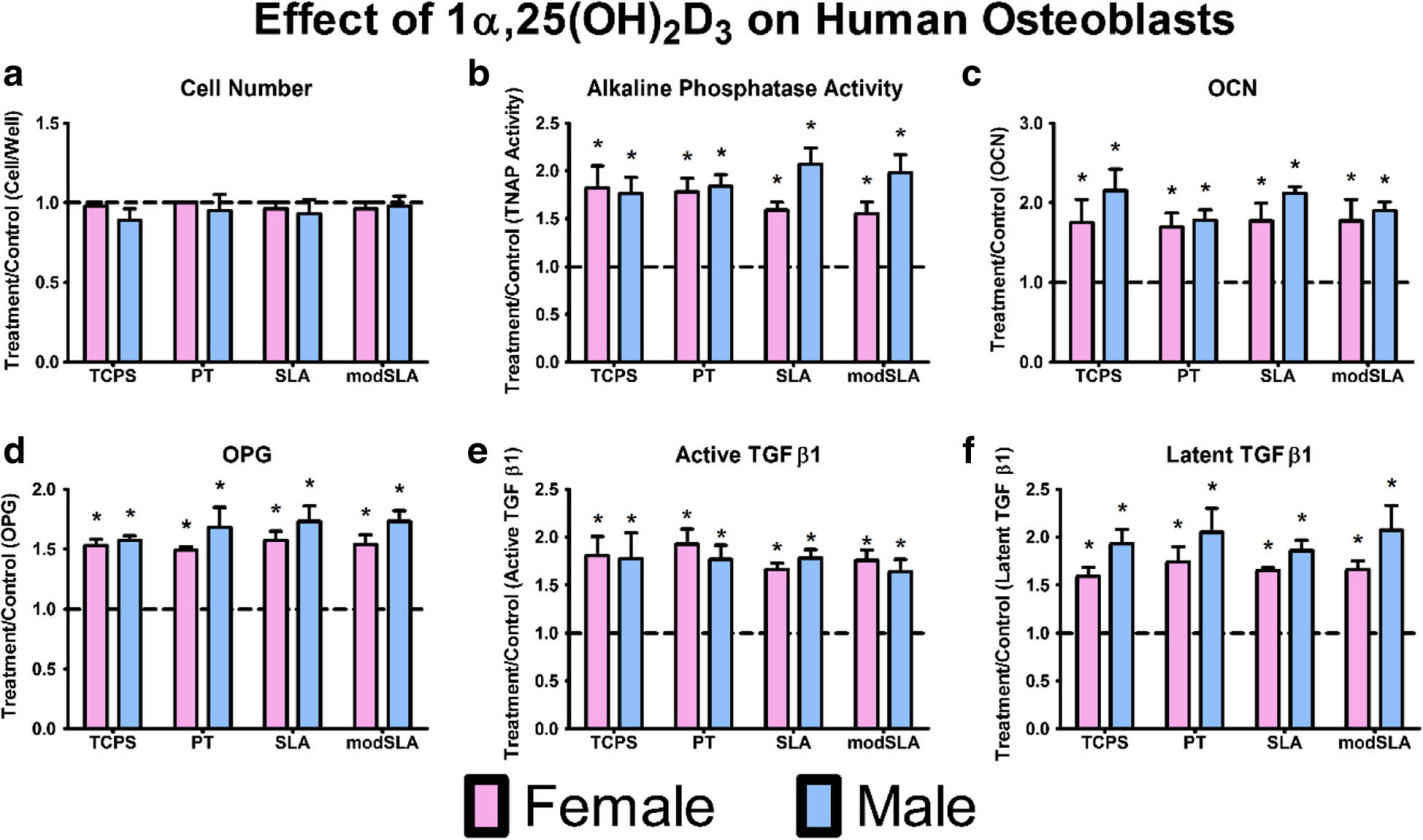
Treatment over control for male and female cells cultured on microstructured Ti surfaces and treated with 1α,25(OH)_2_D_3_ for 24 h at confluence on TCPS. Cell number (**a**), and alkaline phosphatase specific activity in cell lysates was assessed (**b**). Production of osteocalcin (**c**), osteoprotegerin (**d**), and active (**e**) and latent TGF-β1 (**f**) after 24-h fresh medium incubation. *p < 0.05, vs. untreated control per surfaces; •*p* < 0.05, vs. PT; #*p* < 0.05, vs. female cells

Neither female nor male cells altered number due to 1α,25(OH)_2_D_3_ treatment when compared to untreated controls per surface (Fig. 4a). 1α,25(OH)_2_D_3_ increased alkaline phosphatase activity (Fig. 4b), OCN production (Fig. 4c), OPG (Fig. 4d), and active (Fig. 4e) and latent TGF-β1 (Fig. 4f) on all surfaces in male and female osteoblast cultures and to a comparable extent in both. As noted for E_2_, there was considerable variation among donors, although all donors responded to the vitamin D metabolite with comparable fold changes depending on the parameter being examined (Additional file 3: Figure S3A–J; Additional file 2: Figure S2).

## Discussion

The purpose of this study was to examine the role of sexual dimorphism in the responses of male and female osteoblasts to microstructured Ti implants and to determine if sexual dimorphism affected their responses to systemic hormones that regulate bone formation during implant osseointegration. The results show human female and male osteoblasts respond similarly to surface properties. As noted with rat osteoblasts [48], they expressed a more mature phenotype when grown on micro-roughened surfaces (SLA and modSLA) than when they were grown on TCPS or PT, as evidenced by higher levels of alkaline phosphatase activity and OCN production. They also increased local factors OPG and latent TGF-β1 in their conditioned media.

Female and male osteoblasts exhibited differences in average basal characteristics on TCPS, with female cells having higher cellular alkaline phosphatase activity and levels of OCN and both forms of TGF-β1 in their conditioned media compared to male cells. In contrast, male cells had increased basal levels of OPG production, a modulator of osteoclast differentiation and overall activity. Previous studies from our lab have shown that the active form of TGFβ1 signals through autocrine/paracrine pathways to increase the production of OPG [35]. Therefore, female osteoblasts could augment the production of active TGFβ1 to compensate for decreased production of OPG compared to male osteoblasts. Moreover, donor variability among female and male cell populations on each surface was considerable.

Treatment over control analyses provided a clearer assessment of the relative effects of E_2_ or 1α,25(OH)_2_D_3_. Using this approach, we were able to show that female osteoblasts but not male cells responded to E_2_. Although E_2_ stimulated the production of OPG and both forms of TGF-β1 in female cells on all surfaces, the effect of the surface microtexture on response to E_2_ was limited to differentiation; only cell number, alkaline phosphatase activity, and OCN production were sensitive to surface microtexture. In contrast to the response to E_2_, by comparing treatment to control ratios for each parameter on each surface, we did not find differences in the response of male and female human osteoblasts to 1α,25(OH)_2_D_3_ nor did we find a substrate-specific difference in response. This is interesting to note as male rat osteoblasts demonstrated a more robust response to 1α,25(OH)_2_D_3_ compared to female cells [41].

Our previous studies examining the role of substrate microarchitecture on osteoblast response to 1α,25(OH)_2_D_3_ have used cells from male donor rats only, male-female mixed populations, or populations of cells from either male or female rats. In order to be able to use primary cell cultures, none of these studies used cells from single rat donors. Thus, the experimental designs differed from the design used in the present study. Although we have studied human cell response extensively, many of these studies used human MG63 cells, which were originally derived from a male patient [39]. Alternatively, we have used commercial sources to obtain normal human osteoblasts and have used single donors for experiments. In the present study, human male or female cells were collected from three donors of each sex at time of surgery, enabling a more accurate assessment of the potential role of sex differences in osteoblasts during osseointegration.

The donors in this study were all younger than 21 raising the possibility that donor age may have accounted for the difference in human and rat cell response to 1α,25(OH)_2_D_3_ on the microtextured substrates. In many of our previous studies, cells were obtained from sexually mature rats [16]. 1α,25(OH)_2_D_3_ acts on osteoblasts via the vitamin D receptor (VDR) and via a membrane-associated receptor, protein disulfide isomerase A3 (Pdia3) [49], and there may be differences in the expression of these receptors in humans vs. rats as a function of surface topography. However, previous studies to determine if this is the case failed to demonstrate a surface-dependent difference in either receptor, at least in rat cells [15, 50].

Males that lack estrogen receptors (ERs) or aromatase, the enzyme that converts estrogen to testosterone, develop skeletal defects [51], suggesting that E_2_ is important for osseointegration. The fact that E_2_ affected differentiation of female osteoblasts on microstructured Ti but had no effects on the male cells clearly demonstrates that sexual dimorphism exists, but it does not explain why male cells lacked a response to the hormone. Male osteoblasts possess traditional ERs [52], including membrane-associated ERα36 [53], and recent studies show that ERα36 signaling is not functional in male cells [54]. Thus, it is possible that the surface-dependent response to E_2_ is mediated by ERα36 and not by canonical ER-mediated mechanisms. Aromatase activity in the male cells may also be responsible for converting the E_2_ into testosterone, which could result in the non-response seen in male osteoblast donors when treated with E_2_.

Importantly, this study demonstrated that the variability inherent in human donors could impact the consistency of observations, making it imperative to test multiple donors when using primary human osteoblasts for experimentation. Despite the variability, the results confirmed that female and male human osteoblasts respond to Ti surface microstructure with increased maturation and local factor production. Although sex-specific differences in response to substrate microstructure or hydrophilicity of the surface were not detected, the results showed distinct sex differences in response to systemic hormones during osseointegration. Future studies should be examined with increased donor populations to fully define the differences between females and males during implant integration and minimize the risk of subpopulation responses during scientific evaluations.

It is not unsurprising that the male osteoblasts were unresponsive to E_2_ treatment. Previous examination of the effects of E_2_ treatment of adult human donor chondrocytes also showed male cells to be unresponsive [40]. Thus, responsiveness is most likely not age-dependent in males. During mid to late adolescense, bone mass nearly doubles, and bone mass accrual during puberty is a major determinant of overall bone mass throughout adulthood. Differences between male and female overall peak bone mass have been attributed to the later onset of puberty and longer puberty duration in males, with both sexes plateauing at 15–17 years of age. However, studies have shown that E_2_ can alter the thresholds for bone modeling and maintenance to increase and preserve bone mass in females throughout puberty and could be a cause for the variation of response within the female donor population [55, 56]. Some age-dependent changes to ER expression in pre- and postmenopausal osteoblasts are shown with premenopausal osteoblasts exhibiting higher gene expression levels of ERα, and lower levels of ERβ, while postmenopausal osteoblasts are more sensitive to hormones like parathyroid hormone-related peptide (PTHrP) and increase ERβ expression [57]. Male cells are generally not used in studies assessing estrogenic responses, so information on the effects of the hormone in cells from males is limited. Future studies are necessary to determine the impact of age on responsiveness to E_2_. This patient population should consist of age-matched adult males and pre-menopausal females.

The mechanisms regulating bone loss during aging appear to differ between males and females. In females, bone resorption is accelerated by osteoclasts, while in males, age-related bone loss is a result of insufficient new bone formation [58]. The differences in response to E_2_ observed in the present study may also contribute to differences in terminally differentiated osteocytes and osteocyte lacunar density in human T12 vertebral bone. In females, osteocyte differentiation and distribution significantly decrease in an age-dependent manner, but males show no decrease [59]. These differences may also be site specific, as femoral neck bone mineral density decline after hip fracture is not different between men and woman [60].

## Conclusions

The results of the present study revealed that male and female osteoblasts respond to Ti surface micro/meso topography in a comparable manner and are regulated by 1α,25(OH)_2_D_3_ in a comparable way. However, there is clear sexual dimorphism in their response to E_2_ treatment, suggesting that estrogen status and implant surface design may be important variables to consider when using Ti implants in female patients. Future studies should include the use of both male and female cells in order to obtain a better understanding how osseointegration of biomaterials used in orthopedic and dental applications is impacted by sex of the patient.

## Additional files

Additional file 1: **Figure S1.** Donor-specific response for (A) female and (B) male cells cultured on microstructured Ti surfaces and treated with 17β-estradiol for 24 h at confluence on TCPS. Active TGF-β1 after 24-h fresh medium incubation. *p < 0.05, vs. TCPS; ^#^*p* < 0.05, vs. untreated control group per surface. (PDF 270 kb)

Additional file 2: **Figure S2.** Donor-specific response for (A) female and (B) male cells cultured on microstructured Ti surfaces and treated with 1á,25(OH)2D3 for 24 h at confluence on TCPS. Active TGF-â1 after 24-h fresh medium incubation. *p < 0.05, vs. TCPS; ^#^*p* < 0.05, vs. untreated control group per surface. (PDF 266 kb)

Additional file 3: **Figure S3.** Donor-specific response for male and female cells cultured on microstructured Ti surfaces and treated with 1á,25(OH)2D3 for 24 h at confluence on TCPS. Cell number was assessed to measure proliferation (A, F). Alkaline phosphatase-specific activity in cell lysates was assessed (B, G). Production of osteocalcin (C, H), osteoprotegerin (D, I), and latent TGF-â1 (E, J) after 24-h fresh medium incubation. *p < 0.05, vs. TCPS; ^#^*p* < 0.05, vs. untreated control group per surface. (PDF 376 kb)

## Abbreviations

1α,25(OH)_2_D_3_: 1α,25-Dihydroxyvitamin D_3_
BMP2: Bone Morphogenetic Protein 2
E_2_: 17β-Estradiol
MSC: Mesenchymal stem cells
OCN: Osteocalcin
OPG: Osteoprotegerin
PT: Pretreatment
SLA: Sand-blasted Large-grit Acid-etched
TCPS: Tissue Culture Polystyrene
TGF-β1: Transforming Growth Factor Beta 1
Ti: Titanium
VEGF: Vascular Endothelial Growth Factor
