# Supplementary material for: Human osteoblasts exhibit sexual dimorphism in their response to estrogen on microstructured titanium surfaces
Source: Biol Sex Differ. 2018 Jul 3;9:30. doi: 10.1186/s13293-018-0190-x (PMC6029108; doi:10.1186/s13293-018-0190-x)
Supplement: Supplementary file 2 — Figure S2. Donor-specific response for (A) female and (B) male cells cultured on microstructured Ti surfaces and treated with 1á,25(OH)2D3 for 24 h at confluence on TCPS. Active TGF-â1 after 24-h fresh medium incubation. *p < 0.05, vs. TCPS; #p < 0.05, vs. untreated control group per surface. (PDF 266 kb) [file 13293_2018_190_MOESM2_ESM.pdf]

Figure S2

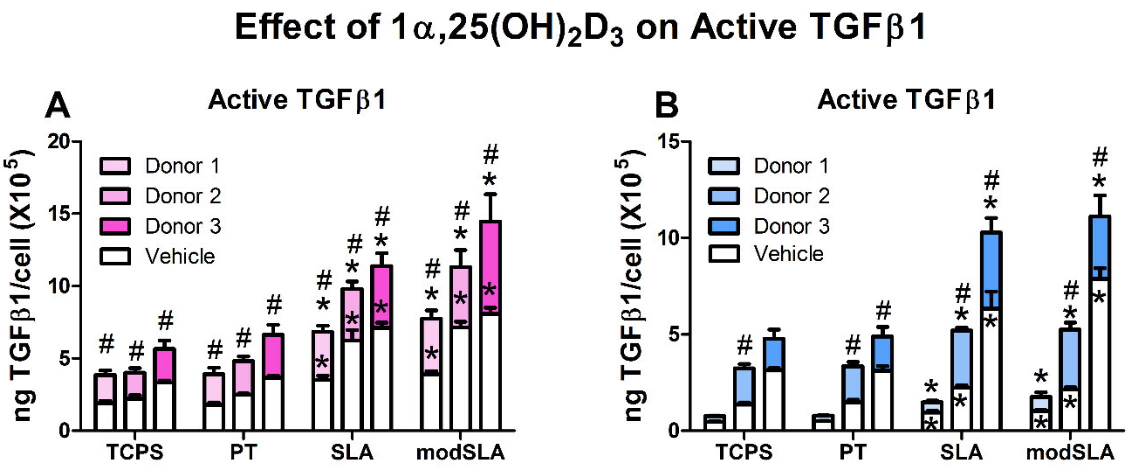

Donor specific response for (A) female and (B) male cells cultured on microstructured Ti surfaces and treated with 1α,25$(OH)_2D_3$ for 24 hours at confluence on TCPS. Active TGF-β1 after 24 hours fresh media incubation. *$p<0.05$, vs. TCPS; #$p<0.05$, vs. untreated control group per surface.
