# Supplementary material for: Human osteoblasts exhibit sexual dimorphism in their response to estrogen on microstructured titanium surfaces
Source: Biol Sex Differ. 2018 Jul 3;9:30. doi: 10.1186/s13293-018-0190-x (PMC6029108; doi:10.1186/s13293-018-0190-x)
Supplement: Supplementary file 3 — Figure S3. Donor-specific response for male and female cells cultured on microstructured Ti surfaces and treated with 1á,25(OH)2D3 for 24 h at confluence on TCPS. Cell number was assessed to measure proliferation (A, F). Alkaline phosphatase-specific activity in cell lysates was assessed (B, G). Production of osteocalcin (C, H), osteoprotegerin (D, I), and latent TGF-â1 (E, J) after 24-h fresh medium incubation. *p < 0.05, vs. TCPS; #p < 0.05, vs. untreated control group per surface. (PDF 376 kb) [file 13293_2018_190_MOESM3_ESM.pdf]

Figure S3

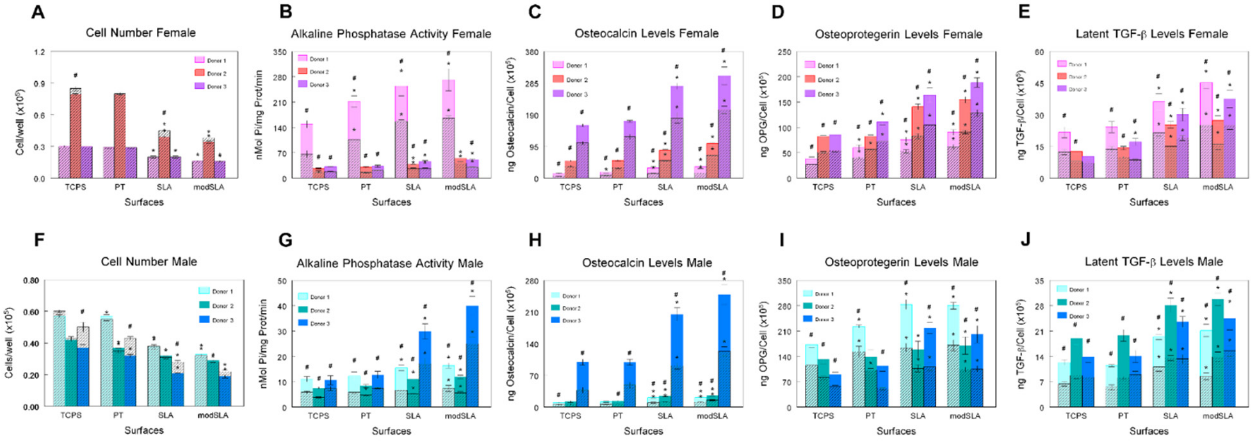

Donor specific response for male and female cells cultured on microstructured Ti surfaces and treated with 1α,25$(OH)_2D_3$ for 24 hours at confluence on TCPS. Cell number was assessed to measure proliferation (A, F). Alkaline phosphatase specific activity in cell lysates was assessed (B, G). Production of osteocalcin (C, H), osteoprotegerin (D, I), and latent TGF-β1 (E, J) after 24 hours fresh media incubation. *$p<0.05$, vs. TCPS; #$p<0.05$, vs. untreated control group per surface.
